# Non-canonical telomere repeat-binding factor 2 control of retinal development

**DOI:** 10.1098/rstb.2025.0339

**Published:** 2026-11-12

**Authors:** Ge Gao, Zhen Xie, Jing Wang, Yilin Ying, Ruochen Pu, Yanping Li, Xiaotuo Zhang, Eric Gilson, Jing Ye

**Affiliations:** ^1^ Department of Geriatrics, Medical Center on Aging of Shanghai Ruijin Hospital, Shanghai Jiaotong University School of Medicine, Shanghai, People's Republic of China; ^2^ Pole Sino-Francais de Recherches en Sciences du Vivant et Genomique, International Research Project ‘Cancer, Aging and Hematology’, Ruijin Hospital, Inserm, CNRS, Shanghai, People's Republic of China; ^3^ The State Key Laboratory of Medical Genomics, Shanghai, People's Republic of China; ^4^ Department of Ophthalmology, Shanghai General Hospital, Shanghai, People's Republic of China; ^5^ University of Côte d'Azur, CNRS, Inserm, Institute for Research on Cancer and Aging, IRCAN, Nice, France; ^6^ Department of Medical Genetics, IHU RespirERA, Nice University Hospital, Nice, France

**Keywords:** telomere repeat-binding factor 2, retinal development, DNA damage response, telomere, ageing

## Abstract

Telomere repeat-binding factor 2 (TRF2) protects chromosome ends but also regulates extra-telomeric chromatin structure and function. Using zebrafish mutants, we show that complete loss of the gene encoding TRF2 (*terfa*) spares early retinal progenitor expansion yet later causes catastrophic Müller glia failure, loss of retinal lamination, genome-wide DNA damage and apoptosis. The eyes of heterozygous zebrafish (*terfa*^+/−^) exhibit preserved gross structure, but Müller glial scaffolding is disrupted, Pax6-derived lineages are reduced and retinal transcriptional programmes are altered without detectable DNA damage, with a subset of phototransduction and regulatory genes remaining mis-regulated in adult eyes. These findings identify TRF2 as a dosage-sensitive, non-canonical regulator of retinal development whose dysfunction reprogrammes retinal transcriptional circuits, suggesting that coupling telomere and genome stability to sensory organ development shapes visual resilience and vulnerability to ageing.

This article is part of the theme issue ‘Developmental circuits in ageing’.

## 1. Introduction

Telomeres, the nucleoprotein structures capping chromosome ends, are essential for preserving genomic integrity during rapid proliferative expansion [1–3]. The shelterin complex, including telomeric repeat-binding factor 2 (TRF2), prevents recognition of natural chromosome ends as double-strand breaks and suppresses inappropriate DNA damage responses (DDR) [4,5]. Notably, TRF2 emerges as a factor with a finely balanced dual role at telomeric and non-telomeric sites, with important implications for development and ageing [1‒3]. In mammals, TRF2 is best known as a core shelterin component that promotes t-loop formation and prevents ATM serine/threonine kinase (Ataxia Telangiectasia Mutated) activation at telomeres and end-to-end fusions [3], thereby preserving telomere integrity during proliferation. However, accumulating evidence indicates that TRF2 also binds extra-telomeric chromatin, where it modulates DNA damage responses and heterochromatin organization [1,4, 6–10]. Zebrafish offer a particularly informative model to study these extra-telomeric functions, as TRF2 retains telomere-binding capacity yet appears to function predominantly as a genome-wide regulator of DNA damage signalling and gene expression, rather than as a predominant telomere capping factor [9].

In this study, we use zebrafish carrying homozygous and heterozygous ablations of the *terfa* gene (encoding TRF2) to study the role of TRF2 during early development. We reveal a prominent effect of *terfa* loss on retinal development, with a disruption of retinal lamination, primarily due to impaired Müller glia morphology and dysregulation of retinal progenitor cell (RPC) proliferation and survival. Notably, no DNA damage occurs upon a half dosage of TRF2 in the heterozygous zebrafish although this leads to important retinal development defects. Overall, these results suggest a role for TRF2 beyond canonical telomere protection in eye development. The TRF2 action during neural development leads us to speculate that coupling telomere and genome stability to sensory systems (here, the eyes) provides an adaptive advantage for environmental resilience.

## 2. Material and methods

### (a) Zebrafish husbandry and ethics statement

The zebrafish Tübingen (Tü) strain was utilized for all experiments. Adult fish were raised and maintained in a systemic recirculating water system under a 14-h light/10-h dark cycle at a constant temperature of 28.5°C, in accordance with international standard protocols. Water quality was strictly monitored to maintain a pH of 7.0–7.5 and a conductivity of 500–800 µS cm^−1^. Fish were fed twice daily with live brine shrimp. Embryos were obtained through natural spawning and cultured in embryo medium (sea salt: 60 mg l^−1^; methylene blue: 0.089 mg l^−1^ to prevent fungal growth). All animal procedures and experimental protocols were approved by and performed in strict accordance with the guidelines of the Institutional Animal Care and Use Committee (IACUC) of Shanghai Jiao Tong University.

### (b) Plasmid construction

The coding sequences (CDS) of zebrafish *terfa* were synthesized (GenScript, China) based on the reference sequence available in the NCBI database. The synthesized cDNA fragment was cloned into the pCS2-P2A-EGFP vector. The resulting constructs, pCS2-*terfa*-P2A-EGFP and pCS2-P2A-EGFP, express *terfa* and the EGFP reporter as separate polypeptides directed by the self-cleaving P2A peptide. The integrity of all constructs was confirmed by Sanger sequencing.

### (c) *In vitro* transcription and microinjection

To generate capped and polyadenylated mRNA, the pCS2-based expression vectors were linearized with *NotI-HF^®^* (R3189S, New England Biolabs) and used as templates for *in vitro* transcription using the mMESSAGE mMACHINE SP6 Transcription Kit (AM1340, Thermo Fisher Scientific, Waltham, MA, USA). The resulting mRNA was purified using the GeneJET RNA purification kit (K0732, Thermo Fisher Scientific) and quantified by spectrophotometry. Microinjections were performed into the yolk of zebrafish embryos at the one-cell stage. The injection volume was calibrated to 0.5–1.0 nl per embryo. Each embryo received approximately 10–20 pg of *terfa* mRNA (full-length) together with 25–100 pg of Tol2 transposase mRNA. Negative control embryos were injected with control mRNA. Successful injection was verified by the ubiquitous expression of EGFP at 24 h post-fertilization (hpf). Embryos exhibiting normal morphology and robust fluorescence were selected and maintained in culturing medium for subsequent experiments. All samples were collected at 72 hpf for experiments afterwards.

### (d) Generation of *terfa*-knockout zebrafish

The *terfa* knockout lines were generated using the CRISPR/Cas9 system targeting the second exon of the *terfa* gene. A specific single-guide RNA (sgRNA) was designed using the ZiFiT Targeter (https://bio.tools/zifit) with the target sequence: 5′-GTGTGGTGGTCAGGCCGG-3′. To construct the sgRNA expression vector, synthesized oligonucleotides were annealed and cloned into a linearized pDR274 backbone following standard molecular cloning procedures. The transcribed sgRNA and Cas9 mRNA were co-injected into one-cell stage zebrafish embryos. These founder animals (F0 generation) were raised to sexual maturity (approx. three months) and subsequently outcrossed with wild-type (WT) zebrafish to produce the F1 generation. Each mutant line was outcrossed for at least six generations and then used in this study. To identify potential indel mutations, genomic DNA was extracted from caudal fin clips of F1 zebrafish and subjected to PCR amplification and Sanger sequencing. The following primers were utilized for genotyping: forward 5′-TTAACCCGCGGTTATCTTCAG-3′ and reverse 5′-CGTCTCCGACATTCACTCAC-3′.

### (e) Tissue processing, paraffin sectioning and haematoxylin and eosin staining

Zebrafish larvae were fixed in 4% paraformaldehyde (PFA) in 0.1 M PBS (pH 7.4) at 4°C for 24 h. After fixation, the specimens were dehydrated through a graded ethanol series (50%, 75% and twice in 100%), cleared in xylene (three changes), and embedded in paraffin wax at 58°C. Serial transverse sections of the head, including the bilateral eyes, were cut at a thickness of 10 µm using a rotary microtome and mounted on poly-L-lysine-coated glass slides. For haematoxylin and eosin (H&E) staining, the sections were first deparaffinized in xylene (two changes, 10 min each) and rehydrated through a descending ethanol series (100%, 95%, 80% and 70%, 5 min each) to distilled water. The nuclei were stained with Harris haematoxylin for 5–8 min, followed by rinsing in running tap water for 10 min. Differentiation was performed in 1% acid alcohol for 3 s and ‘bluing’ in 0.1% sodium bicarbonate for 1 min. After a brief wash, the cytoplasm was counterstained with 0.5% eosin Y for 2 min. Finally, the sections were dehydrated through an ascending ethanol series, cleared in xylene and permanently mounted with neutral resin. Representative images were captured using a Nikon Eclipse Ni-U upright microscope.

### (f) Retinal layer thickness and cell quantification

Paraffin-embedded eyes were sectioned along the vertical axis following standardized orientation during embedding. To minimize artefactual distortion caused by oblique sectioning, only mid-meridional sections were selected for analysis. A section was included only when the optic nerve head and optic nerve tract were clearly visible and continuous with the retina, ensuring comparable anatomical alignment across samples.

For retinal layer thickness measurements, regions of interest (ROIs) were defined in comparable regions of the central retina. Layer thickness was measured as the distance between anatomically defined boundaries using ImageJ software. Cell quantification was performed across the entire retinal section to capture global changes in cell number. All quantitative analyses were conducted in a blinded manner where applicable.

### (g) Cryo-sectioning and immunofluorescence

For cryo-sectioning, zebrafish larvae were fixed in 4% PFA at 4°C overnight. To ensure cryoprotection and prevent ice crystal artefacts, fixed samples were dehydrated in 30% sucrose in PBS at 4°C until they reached equilibrium (sedimentation). The specimens were then embedded in optimal cutting temperature (OCT) compound (Tissue-Tek) and rapidly frozen at -80°C. Serial transverse sections (10 µm) were obtained using a Leica cryostat (CM1950) and mounted on SuperFrost Plus slides. For immunofluorescence staining, the sections were post-fixed in 4% PFA for 30 min at room temperature, followed by three washes in PBS. To facilitate antibody penetration and minimize non-specific binding, sections were permeabilized and blocked in a buffer containing 0.5% Triton X-100 and 3% foetal bovine serum (FBS) in 1×PBS for 1 h at room temperature. The sections were then incubated with primary antibodies (details provided in table S1, supplementary material) at 4°C in a humidified chamber overnight. After three washes with PBST (0.1% Tween-20 in PBS), the sections were incubated with corresponding Alexa Fluor-conjugated secondary antibodies (Invitrogen, 1 : 500) for 2 h at 37°C. Nuclei were counterstained with 1×DAPI for 10 min. Finally, the slides were mounted with an anti-fade mounting medium. Confocal imaging was performed using a Zeiss LSM 980 confocal laser scanning microscope equipped with high-sensitivity spectral detectors. Images were processed and analysed using ZEN (blue edition).

### (h) Peptide nucleic acid fluorescence *in situ* hybridization

To detect telomeric DNA and specific proteins simultaneously, a combined protocol of peptide nucleic acid fluorescence *in situ* hybridization (PNA-FISH) and immunofluorescence was employed on cryosections. Briefly, the sections were post-fixed in 4% PFA containing 0.5% Triton X-100 for 15 min to enhance probe accessibility. Following fixation, sections were dehydrated through an ascending ethanol series (50%, 75% and 100%) for 5 min each and air-dried. The DNA was denatured by incubating the slides in a solution of 70% formamide, 10 mM Tris (pH 7.2) and 1% blocking reagent (Roche) at 85°C for 5–6 min. Immediately following denaturation, the PNA probe ((TTAGGG)*_n_*] was hybridized to the sections at room temperature for at least 2 h in a humidified chamber. To remove non-specific binding, the slides were subjected to stringent washes: first in a 70% formamide and 10 mM Tris (pH 7.2) solution for 30 min, followed by a buffer containing 150 mM NaCl and 50 mM Tris (pH 7.5) for 15 min. Subsequently, sections were processed for immunofluorescence as mentioned above.

### (i) Ionizing radiation treatment

Eight days post-fertilization (dpf) larvae were placed in a 15 ml centrifuge tube containing a minimal volume of culturing medium to ensure uniform exposure. Irradiation was performed using an X-ray irradiator (Gammacell 1000) at a total dose of 6147 cGy, delivered continuously over a duration of 20 min (approximate dose rate of 307.35 cGy min^−1^). Following irradiation, the larvae were immediately transferred to fresh culturing medium and maintained at 28.5°C for 1 h to allow for the robust recruitment of DNA damage response proteins prior to fixation in 4% PFA. Subsequent processing was performed as described in the previous sections.

### (j) RNA extraction and real time-qPCR

Total RNA was extracted from zebrafish embryos and tissues using TRIzol reagent (Ambion) and reverse transcribed into cDNA using the PrimeScript RT reagent kit (TaKaRa) according to the manufacturers’ protocols. RT-qPCR analysis was performed on a QuantStudio Dx Real-Time PCR Instrument (ABI) using SuperReal PreMix Plus (TianGen). The sequences of all primers used in this study are listed in supplementary material, table S1.

### (k) Single-nucleus RNA-seq and data analysis

Brain tissues from *terfa*^+/+^, *terfa*^+/−^ and *terfa*^−/−^ larvae (3 dpf) were used for single-nucleus RNA sequencing (snRNA-seq) analysis. The heads were dissected just anterior to the spinal cord. Tissue samples were surgically isolated and snap-frozen in liquid nitrogen for subsequent nucleus isolation. Nuclei were isolated and purified as previously described with minor modifications. Briefly, frozen tissues were homogenized in NLB buffer containing 250 mM sucrose, 10 mM Tris-HCl, 3 mM MgAc₂, 0.1% Triton X-100 (Sigma-Aldrich, USA), 0.1 mM EDTA and 0.2 U µl^−1^ RNase inhibitor (Takara, Japan). Gradient sucrose solutions were used to further purify nuclei. The concentration of nuclei was adjusted to approximately 1000 nuclei µl^−1^. Single-nucleus RNA-seq libraries were generated using the 10x Genomics Chromium Controller and Chromium Single Cell 3′ v3 reagent kits (10x Genomics, Pleasanton, CA). Nuclei suspensions were loaded into each channel to generate gel bead-in-emulsions (GEMs). Following reverse transcription, GEMs were broken and barcoded cDNA was purified and amplified. The amplified cDNA was fragmented, A-tailed, adaptor-ligated and subjected to index PCR amplification.

Final libraries were quantified using the Qubit High Sensitivity DNA assay (Thermo Fisher Scientific), and size distribution was assessed using a high sensitivity DNA chip on a Bioanalyzer 2200 (Agilent). Sequencing was performed on an Illumina platform using a 150 bp paired-end configuration. snRNA-seq data were processed and analysed using the NovelBrain Cloud Analysis Platform (www.novelbrain.com) by NovelBio Co., Ltd.

### (l) RNA-seq and data analysis

All retinal tissue from six-month-old (6 mpf) t*erfa*^+/+^ and *terfa*^+/−^ zebrafish was used for RNA sequencing analysis. Total RNA extracted from zebrafish retinas was submitted to BGI (China) for library construction and sequencing. Sequencing was performed on the Illumina HiSeq X Ten platform in a 150 bp paired-end mode, generating more than 40 million raw reads per sample. The experiment was conducted with three biological replicates for each group. For data processing, raw reads were filtered using SOAPnuke with default settings to remove adaptors and low-quality reads. The remaining clean reads were trimmed to 100 bp for downstream analysis. Sequence alignment to the zebrafish reference genome (zv10) was performed using STAR (v2.6.1d) with the parameter—quantMode GeneCounts. Differential expression analysis between the *terfa* mutant and wild-type groups was subsequently performed using the DESeq2 R package.

### (m) Statistical analysis

All statistical analyses were performed using GraphPad Prism 10.0 (GraphPad Software, San Diego, CA, USA). Quantitative data are presented as the mean ± standard error of the mean (s.e.m.). The number of independent biological replicates (*n*), representing the number of individual eyes or cells analysed, is specified in the corresponding figure legends. Data were assessed for normality prior to statistical analysis. For normally distributed data, parametric tests were applied as described below; otherwise, appropriate non-parametric tests were used. For comparisons between two experimental groups, a two-tailed Student's *t*-test was employed. For multiple group comparisons involving a single independent variable, ordinary one-way ANOVA followed by Tukey's post-hoc test was performed to determine specific pairwise significances. In experiments involving two independent factors (e.g. genotype and mRNA supplementation), two-way ANOVA was utilized. Statistical significance was pre-defined as *p* < 0.05. Significance levels are denoted in the figures as follows: *p < 0.05, **p < 0.01, ***p < 0.001, ****p < 0.0001.

## 3. Results

### (a) Telomere repeat-binding factor 2 loss-of-function mutation induces neural developmental failure in the retina

Retinal neurogenesis is a highly coordinated process in which multipotent RPCs sequentially generate diverse neuronal and glial lineages from a common progenitor pool [11]. In vertebrates, this process begins during embryogenesis and extends into early postnatal stages, ultimately giving rise to a laminated retinal architecture composed of distinct strata: the photoreceptor-enriched outer nuclear layer (ONL), the inner nuclear layer (INL) containing bipolar and amacrine cells, and the retinal ganglion cell layer (GCL) [9,10] (figure 1A).

**Figure 1.**
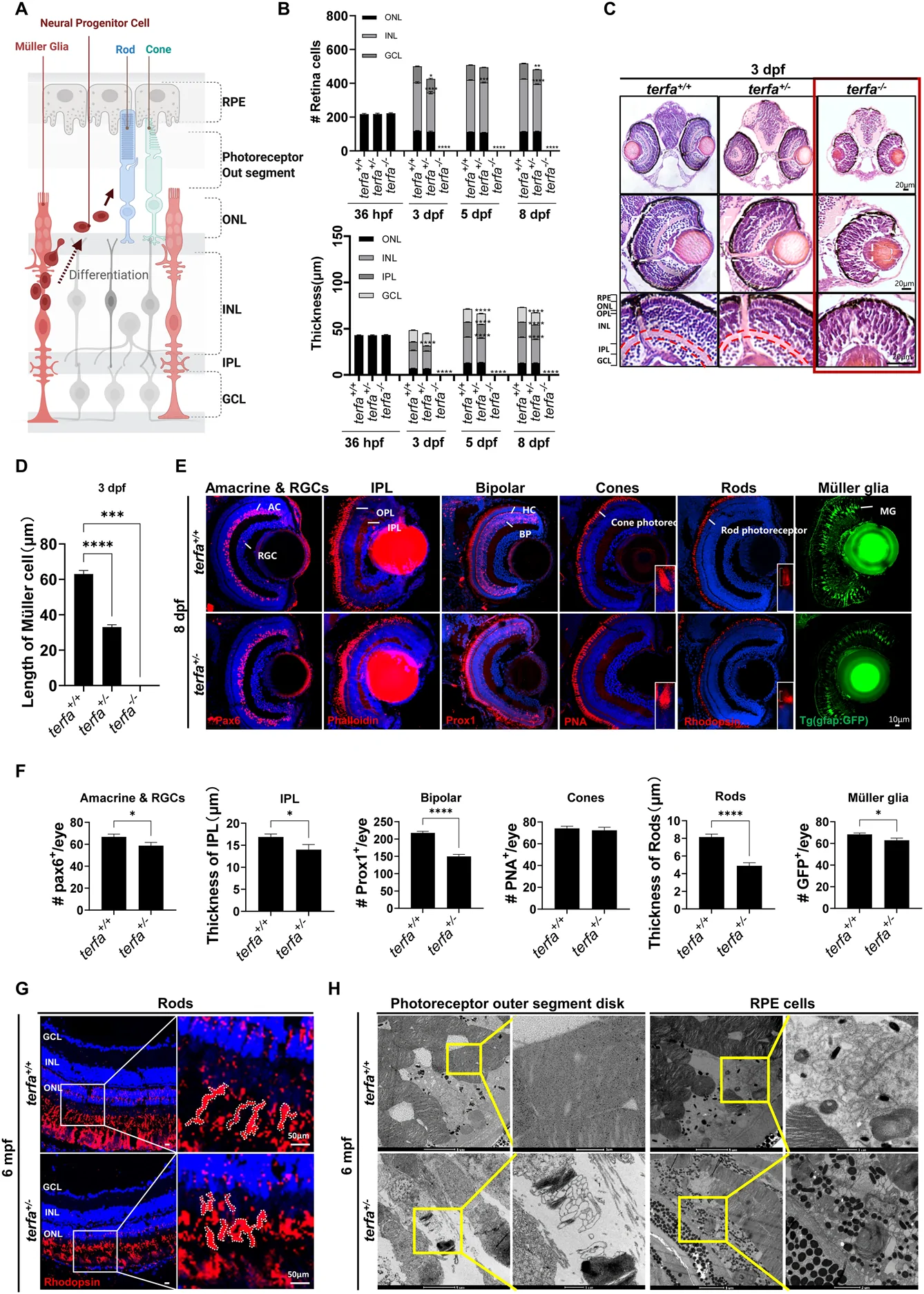
TRF2 ablation induces neural developmental failure in the retina. (A) Schematic diagram of zebrafish retinal architecture and a proposed differentiation model. Retinal layers are labelled from outer to inner as: retinal pigment epithelium (RPE), photoreceptor outer segment, outer nuclear layer (ONL), inner nuclear layer (INL), inner plexiform layer (IPL) and ganglion cell layer (GCL). Müller glia (red) are depicted as radial cells spanning multiple layers, with cell bodies located in the INL. Neural progenitor cells (dark red) are shown migrating from the INL toward the ONL and differentiating into photoreceptor lineages, including rods (blue) and cones (teal). (B) Total retinal cell number and retinal thickness quantified at 36 hpf, 3 dpf, 5 dpf and 8 dpf across *terfa*^+/+^, *terfa*^+/−^ and *terfa*^−/−^. At 36 hpf (pre-stratification), bars show total cell counts and total retinal thickness (*n* = 36, 32, 36 eyes for *terfa*^+/+^, *terfa*^+/−^, *terfa*^−/−^, respectively). At 3 dpf (*n* = 61 eyes for *terfa*^+/+^, *n* = 34 for *terfa*^+/−^), 5 dpf (*n* = 43, 35) and 8 dpf (*n* = 72, 81), stacked bars indicate layer-specific contributions: cell counts partitioned into ONL/INL/GCL; thickness partitioned into ONL/INL/IPL/GCL. *terfa*^−/−^ values were set to 0 at 3 dpf for layer-specific analysis due to absent lamination and were omitted at 5/8 dpf due to larval lethality. (C) Representative transverse H&E sections of 3 dpf retinas (*terfa*^+/+^, *terfa*^+/−^, *terfa*^−/−^) with magnified ROIs and annotated layers (RPE, ONL, OPL, INL, IPL, GCL); dashed lines/arrows indicate measurement intervals. OPL, outer plexiform layer. Scale bar, 20 µm. (D) Quantification of Müller cell length in 3 dpf retina (GCL basal end-feet to ONL apical processes). Up to 10 Müller cells were measured per eye (total *n* = 46 cells for *terfa*^+/+^; *n* = 146 cells for *terfa*^+/−^). *terfa*^−/−^ was counted as 0. (E) Representative 8 dpf transverse retinal sections (*terfa*^+/+^, *terfa*^+/−^) stained with cell-type/layer markers and DAPI: Pax6 (amacrine & RGCs), phalloidin (IPL), Prox1 (bipolar), PNA (cones, insets show outer segments), rhodopsin (rods, insets show outer segments) and Tg(gfap:GFP) for Müller glia. Scale bar, 10 µm. (F) Quantification of retinal cell numbers or layer thickness corresponding to the markers/structures in (E) (*n* = 20 eyes per genotype). Data are presented as mean ± s.e.m. Statistical analyses were performed using unpaired two-sided *t*-tests (*p < 0.05, **p < 0.01, ***p < 0.001, ****p < 0.0001). (G) Representative immunofluorescence images of retinal sections from 6 mpf *terfa*^+/+^ and *terfa*^+/−^ zebrafish stained with anti-rhodopsin (rod photoreceptor outer segments, red) and counterstained with DAPI (nuclei, blue). Left panels show low-magnification views, with boxed regions displayed at higher magnification in the right panels. Dashed outlines indicate individual rod outer segments. Scale bar, 50 µm. (H) Representative transmission electron microscopy (TEM) images of retinal ultrastructure from 6 mpf *terfa*^+/+^ and *terfa*^+/−^ zebrafish. Left panels: Photoreceptor outer-segment disc membranes: low-magnification views with boxed regions shown at higher magnification. Right panels: Retinal pigment epithelium (RPE): low-magnification views with boxed regions shown at higher magnification. Scale bars: 5 µm in low-magnification images and 1 µm in high-magnification images.

At 36 hpf, a developmental stage preceding the onset of retinal stratification, H&E staining revealed that retinal morphology was comparable across all three genotypes (supplementary material, figure S1A). Quantitative analysis at this stage showed no significant differences in either total retinal cell number or overall retinal thickness among *terfa*^+/+^, *terfa*^+/−^ and *terfa*^−/−^ larvae (figure 1B), indicating that TRF2 is not required for the initial expansion of the primitive progenitor pool at this stage.

However, by 3 dpf, *terfa*^−/−^ mutants exhibited a catastrophic failure in retinal lamination, associated with striking microphthalmia and a complete loss of stratified architecture (figure 1B,C). Histologically, the neural retina in *terfa*^−/−^ larvae was reduced to an unstructured cellular mass, devoid of discernible ONL, INL or GCL layers. In our quantitative datasets, both cell number and layer thickness were recorded as zero for *terfa*^−/−^ mutants at 3 dpf, as the absence of laminar organization precluded meaningful measurements (figure 1B). Confocal immunofluorescence analysis using GFAP (glial fibrillary acidic protein) and GS (glutamine synthetase) markers revealed that *terfa*^−/−^ Müller glia displayed fragmented, truncated morphologies and a complete loss of radial integrity at 3 dpf (figure 1D; supplementary material, figure S1B). This phenotype persisted at 5 and 8 dpf, at which points all *terfa*^−/−^ larvae had succumbed, consistent with previously documented developmental lethality [9,12] (supplementary material, figure S1C).

While *terfa*^+/−^ larvae could form retinal layers, they displayed clear architectural deficits relative to wild-type siblings. Specifically, *terfa*^+/−^ retinas exhibited significant reductions in both cell number and layer thickness at 3, 5 and 8 dpf (figure 1B,C; supplementary material, figure S1A). These structural abnormalities were closely linked to disruption of the Müller glial scaffold: quantitative assessment showed a significant reduction in Müller process length in *terfa*^+/−^ larvae compared with *terfa*^+/+^ controls across all time points (figure 1D,E; supplementary material, figure S1B,C). This disruption of the glial network was accompanied by impaired differentiation of the retinal lineages in *terfa*^+/−^ larvae at 8 dpf: while cone photoreceptors (PNA⁺) were unaffected, there were significant reductions in amacrine and retinal ganglion cells (Pax6⁺), bipolar cells (Prox1⁺) and rod photoreceptor length (rhodopsin⁺) relative to *terfa*⁺^/^⁺ controls (figure 1E,F). Moreover, the number of Müller glial cells in Tg(gfap:GFP) lines was significantly decreased (figure 1E,F). When the mRNA of *terfa* was injected into the fish eggs, the retinal deficit phenotype was no longer observed, eliminating non-targeted effects of *terfa* ablation (supplementary material, figure S1D,E).

To uncover the underlying cellular defect, we performed BrdU birth-dating assays to trace progenitor fate (pulse at 18 hpf, analysis at 3 dpf). Immunofluorescence revealed extensive colocalization of Pax6 with BrdU-labelled nuclei at this early stage, confirming Pax6 as a robust marker of the direct progeny of early proliferative RPCs (supplementary material, figure S1F). Quantitative analysis showed a significant reduction in BrdU⁺/Pax6⁺ double-positive cells in *terfa*^+/−^ larvae compared with wild-type controls (supplementary material, figure S1F), indicating that the TRF2 dosage is critical for efficient expansion or maintenance of the early Pax6-positive neurogenic pool.

In adult fish, retinal alterations persisted in heterozygous zebrafish at six months post-fertilization (6 mpf): the photoreceptor outer segments became progressively thinner and more disorganized with age as visualized by rhodopsin immunofluorescence staining (figure 1G). Transmission electron microscopy showed that WT retinas had normal photoreceptor outer segments and RPE cells, whereas retinas from heterozygous zebrafish displayed swollen outer segment disc membranes and accumulation of dense pigment material (figure 1H).

Collectively, these data identify TRF2 as a critical upstream regulator necessary for maintaining the Müller glial scaffold and enabling proper lamination and differentiation of the neural retina. The developmental deficit caused by *terfa* loss, already apparent upon gene haploinsufficiency albeit of lesser intensity, appears to be specific to a relatively late developmental stage (3 dpf) and to a particular cell type (Müller glia). Therefore, this delayed phenotype, even in *terfa*^−/−^ zebrafish, indicates that TRF2 mutation has no lethal effect during the first 3 days of retinal development, i.e. after a period of rapid cell expansion. Together with the selective depletion of retinal lineages, these results suggest a role of TRF2 in retinal development that cannot be merely explained by its roles in DNA damage response and telomere protection and that persists in adulthood.

### (b) Telomere repeat-binding factor 2 dosage separates genome instability from neurogenic defects in the retina

To determine whether retinal collapse in *terfa* mutants reflects telomere-specific dysfunction or broader genomic instability, we examined the spatiotemporal pattern of γH2AX activation. At 3 dpf, confocal imaging revealed a large number of nuclear γH2AX foci, roughly 50% of them colocalizing with telomeres throughout the *terfa*^−/−^ retina (figure 2A,B). This pattern indicates that a full ablation of *terfa* triggers DNA damage signalling both genome-wide and at telomere DNA. This DNA damage coincided with a wave of programmed cell death: quantification of activated Caspase-3 and TUNEL-positive nuclei at 3 dpf revealed a massive increase in apoptosis in *terfa*^−/−^ larvae (supplementary material, figure S2E,F).

**Figure 2.**
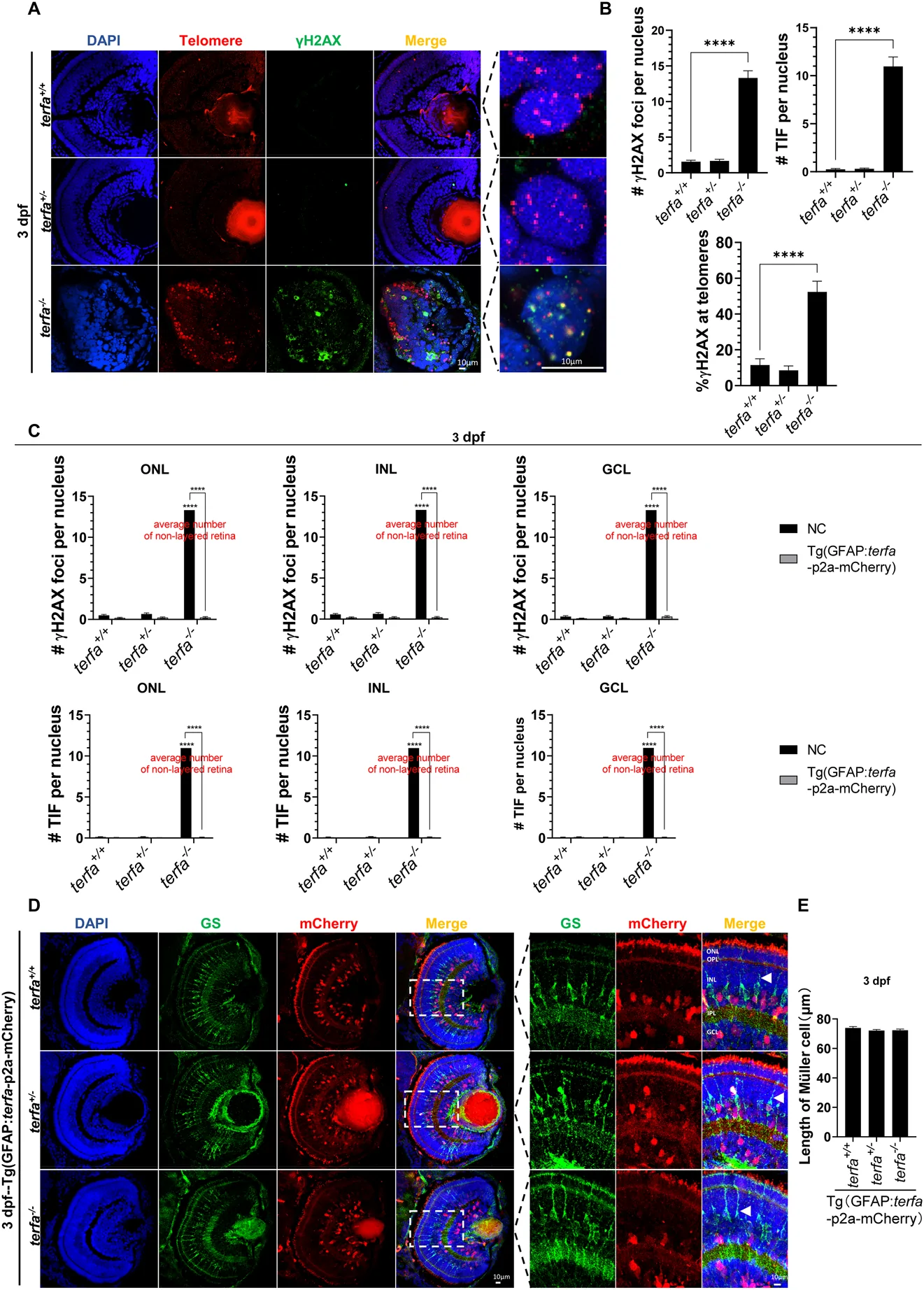
TRF2 ablation leads to genome-wide DDR with no-telomere-specificity in the retina. (A) Representative confocal images of 3 dpf transverse retinal sections stained for γH2AX and telomeres with DAPI counterstain (blue). Images are shown for *terfa*^+/+^, *terfa*^+/−^ and *terfa*^−/−^. Left panels show retinal overviews; dashed boxes/lines mark regions magnified in the right panels. Scale bar, 10 µm. (B) Quantification of γH2AX foci, telomere dysfunction-induced foci (TIFs) and γH2AX at telomeres per nucleus in total retinal cells at 3 dpf. For each eye, 3–5 cells were randomly selected for analysis (*n* = 50 cells for *terfa*^+/+^, *n* = 53 for *terfa*^+/−^, *n* = 32 for *terfa*^−/−^). Data are presented as mean ± s.e.m. Statistical analyses were performed using ordinary one-way ANOVA followed by Tukey's multiple comparisons test (*p < 0.05, **p < 0.01, ***p < 0.001, ****p < 0.0001). (C) Layer-specific quantification of γH2AX foci and TIFs per nucleus in 3 dpf retinas from NC and Tg(GFAP:*terfa*-p2a-mCherry) groups across *terfa*^+/+^, *terfa*^+/−^ and *terfa*^−/−^ larvae within the GCL, INL and ONL. In *terfa*^−/−^ NC retinas, severe disruption of retinal lamination precluded layer-specific analysis; values therefore represent the average number of foci or TIFs per nucleus quantified from non-layered retinal regions. In contrast, Tg(GFAP:*terfa*-p2a-mCherry) partially restored retinal lamination in *terfa*^−/−^ larvae, enabling layer-specific quantification. The figure therefore shows the changes in γH2AX foci and TIF numbers before and after Müller glia-targeted *terfa* rescue. For each group, 5–10 cells were randomly selected per eye, with a total of *n* = 55 cells per group. (D) Representative confocal images of 3 dpf transverse retinal sections from Tg(GFAP:*terfa*-p2a-mCherry) zebrafish, shown for *terfa*^+/+^, *terfa*^+/−^ and *terfa*^−/−^. Sections were immunostained for GS (green) and counterstained with DAPI (blue); mCherry (red) indicates transgene-derived fluorescence. Left panels show low-magnification views of whole retinas, with dashed boxes indicating regions magnified in the right panels. High-magnification images display GS, mCherry and merged signals. Arrowheads indicate representative Müller glial processes. Scale bar, 10 µm. (E) Quantification of Müller cell length in 3 dpf Tg(GFAP:*terfa*-p2a-mCherry) zebrafish retinas (GCL basal end-feet to ONL apical processes) across *terfa*^+/+^, *terfa*^+/−^ and *terfa*^−/−^. Data are presented as mean ± s.e.m. (*n* = 118 cells for *terfa*^+/+^, *n* = 119 for *terfa*^+/−^, *n* = 122 for *terfa*^−/−^). Statistical analyses were performed using ordinary one-way ANOVA followed by Tukey's multiple comparisons test. (ns, not significant.)

Although heterozygous zebrafish exhibit clear retinal developmental defects, no overt DNA damage signalling activation can be detected: *terfa*^+/−^ larvae showed no significant increase in γH2AX foci, telomere deprotection or Caspase-3 activity in the cells of the different retinal layers at 3 dpf (figure 2C; supplementary material, figure S2A,C–E). As a control, a large number of γH2AX foci were triggered upon 8 dpf zebrafish irradiation (supplementary material, figure S2C). To test the specific requirement for TRF2 in Müller glia, we generated a transgenic line (Tg(GFAP:*terfa*-p2a-mCherry)) enabling glia-specific expression of TRF2 (figure 2D) [9]. Targeted restoration of TRF2 in GFAP-positive cells fully rescued the Müller glial process defect in both *terfa*^−/−^ and *terfa*^+/−^ larvae, restoring process length to a level comparable to that of wild-type controls (figure 2E), supporting that TRF2 function within Müller glia is required to maintain radial scaffold integrity. In addition, restoration of TRF2 in Müller glia reduced the elevated DNA damage response observed in *terfa*^−/−^ retinas (figure 2C; supplementary material, figure S2B).

Together, these data suggest that in *terfa*^−/−^ mutants, severe developmental abnormalities are linked to genome-wide DDR and massive cell death. By contrast, the developmental defects observed in *terfa*^+/−^ larvae indicate a role of TRF2 in retinal neurogenesis by regulating RPC functions, and particularly Müller glial differentiation, through a mechanism that appears to be independent of its canonical DNA damage-protective role, whether at telomeres or genome-wide.

### (c) Retinal transcriptional reprogramming following telomere repeat-binding factor 2 mutation

Next, we examined the transcriptional consequences of TRF2 loss. To this end, we analysed snRNA-seq data from 3 dpf zebrafish brain of wild type, heterozygous and homozygous zebrafish and assessed differential gene expression in cluster 3, which is enriched in retinal genes (supplementary material, figure S3A; differentially expressed genes or DEGs listed in supplementary material, table S2). The up- or down-regulation of selected genes was validated by RT-qPCR on brain RNA (supplementary material, figure S3B). In both homozygous and heterozygous mutants, genes involved in phototransduction were strongly decreased (figure 3A), indicating that the retinal phenotype, even in heterozygous zebrafish, is associated with a retinal gene-specific transcriptional signature.

**Figure 3.**
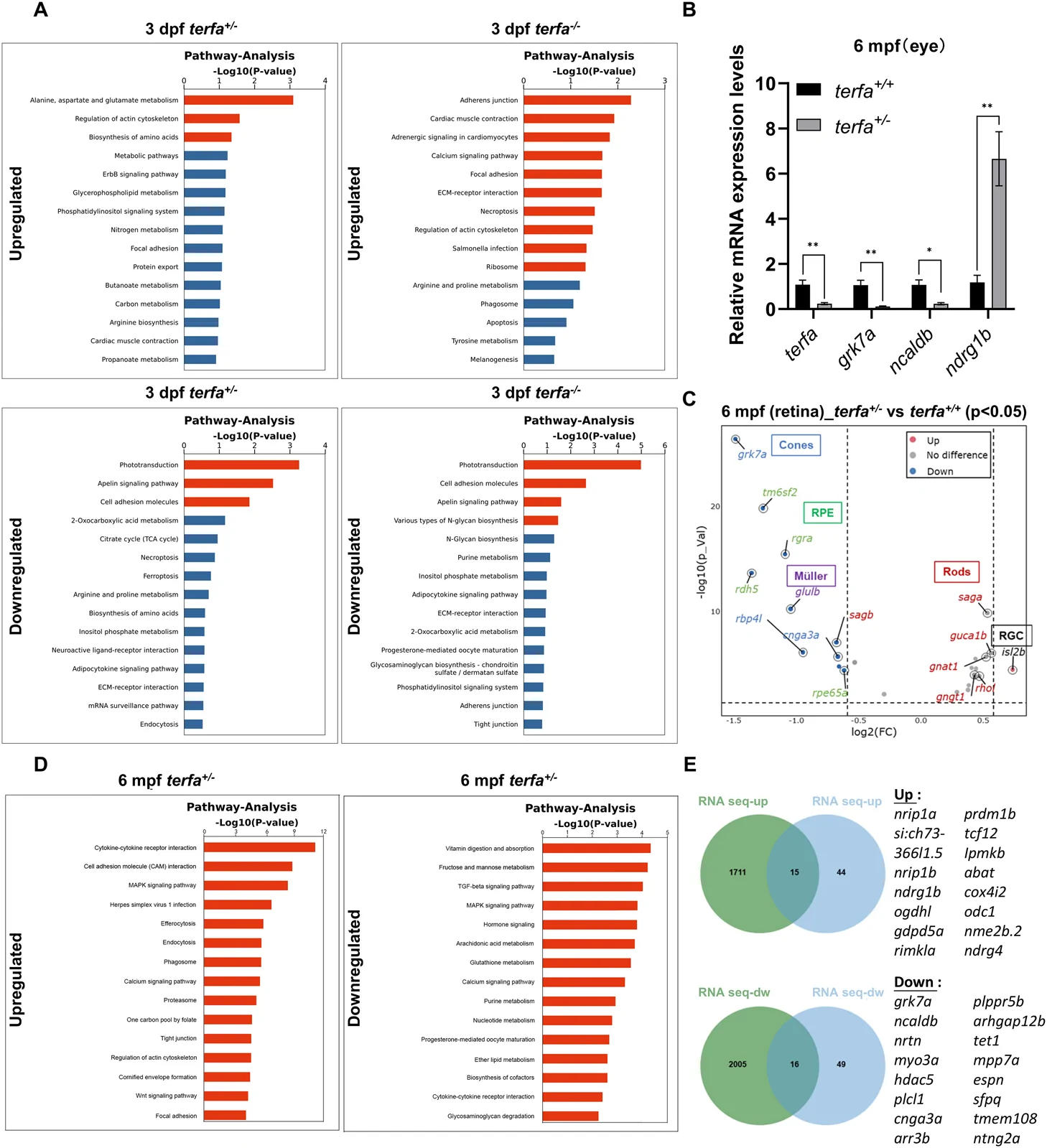
TRF2 ablation leads to retinal transcriptional reprogramming. (A) KEGG pathway enrichment of upregulated and downregulated genes in *terfa*^+/−^ mutants versus *terfa*^+/+^ controls. Panels show snRNA-seq-based enrichment for retinal Cluster 3 at 3 dpf (*terfa*^+/−^ versus *terfa*^+/+^ and *terfa*^−/−^ versus *terfa*^+/+^, respectively). The *x*-axis indicates significance (−log_10_ *p* value); red bars denote significant pathways (*p* < 0.05) and blue bars denote non-significant pathways. (B) RT–qPCR validation of selected candidates (*terfa*,* grk7a*,* ncaldb*,* ndrg1b*) in eye tissues from 6 mpf *terfa*^+/+^ and *terfa*^+/−^ zebrafish, presented as relative mRNA expression (mean ± s.e.m.). Statistics: *p < 0.05, ** *p* < 0.01. (C) Volcano plot of differentially expressed genes in retinas from 6 mpf *terfa*^+/−^ versus *terfa*^+/+^ zebrafish. The *x*-axis indicates log_2_ fold change (FC) and the *y*-axis indicates −log_10_(*p* value). Point colours (inset legend): red, significantly upregulated (Up; *p* < 0.05); blue, significantly downregulated (Down; *p* < 0.05); grey, not significant (No difference). Gene label font colours (inset legend): red, rods; blue, cones; green, retinal pigment epithelium/RPE; purple, Müller glia; black, retinal ganglion cells/RGC. (D) Panels show KEGG pathway enrichment based on bulk RNA-seq of 6 mpf retinas (*terfa*^+/−^ versus *terfa*^+/+^). The *x*-axis indicates significance (−log_10_*p* value); red bars denote significant pathways (*p* < 0.05). (E) Left: Venn diagrams showing the overlap of significantly upregulated (top) and downregulated (bottom) DEGs between bulk RNA-seq and snRNA-seq datasets, used to define candidate genes for validation. Right: lists of overlapping upregulated (top) and downregulated (bottom) genes.

We then analysed bulk RNA-seq from retinas of 6 mpf wild-type and heterozygous zebrafish. A distinct transcriptional programme between the two genotypes was revealed by principal component analysis (supplementary material, figure S3C) and the corresponding DEGs are listed in the supplementary material, table S3. The differential expression of a subset of these DEGs was confirmed by qPCR on eye tissue (figure 3B). Key marker genes of several retinal cell types were either up- or down-regulated (figure 3C). Pathway enrichment analysis pointed to a predominant immunity/inflammation signature, suggesting that 6 mpf heterozygous eyes are inflammatory (figure 3D). The KEGG enrichment of immune/inflammatory pathways in 6 mpf *terfa*^+/−^ retinas (figure 3D) prompted us to validate a key effector molecule, interleukin-6 (IL-6), whose upregulation is consistently linked to chronic retinal inflammation and photoreceptor degeneration. Immunofluorescence analysis revealed a significant, layer-specific increase in IL-6 protein levels within the rod photoreceptors and their synaptic terminals in the outer plexiform layer (OPL) of t*erfa*^+/−^ adults (supplementary material, figure S3D,E). This IL-6 elevation is accompanied by the progressive thinning and disorganization of rod outer segments observed by rhodopsin staining and TEM (figure 1G,H), suggesting that TRF2 deficiency-induced transcriptional reprogramming culminates in a localized, neuroinflammatory microenvironment that actively contributes to structural and functional decline.

We next asked whether a transcriptional signature persisted during development in heterozygous zebrafish. To address this, we compared cluster 3 DEGs with those from the 6 mpf retinal RNA-seq dataset. Among the common genes, 16 were down-regulated and 15 were up-regulated (figure 3E; supplementary material, table S4, which lists their functional descriptions). The 16 down-regulated genes are mostly neuronal and strongly associated with cone photoreceptors and their synapses, whereas the 15 up-regulated genes are consistent with altered neuronal differentiation/identity driven by transcriptional regulators (supplementary material, table S4). In summary, these results argue that the retinal phenotype of heterozygous zebrafish that persists during development reflects an unbalanced reprogramming of retinal neuronal circuits leading to eye inflammation in adult zebrafish.

## 4. Discussion

TRF2 emerges from this work as a regulator of retinal development whose function cannot be explained solely by its canonical role in telomere capping and DNA damage suppression. In zebrafish, TRF2 (encoded by the *terfa* gene) has been shown to act as a genome-maintenance and transcriptional regulator in glial cells that is specifically required for neurodevelopment and normal ageing [9]. Our data extend these findings to the retina, revealing a dosage-sensitive requirement for TRF2 in Müller glial integrity, retinal lamination and lineage specification, and providing evidence that TRF2 dysfunction during development imprints a long-lasting transcriptional signature that persists into adult life.

The temporal and cellular selectivity of the retinal phenotype argues for a non-canonical developmental role of TRF2. At 36 hpf, *terfa*^+/+^*, terfa*^+/−^ and *terfa*^−/−^ retinas are indistinguishable in size and cellularity, indicating that early expansion of the primitive retinal progenitor pool proceeds normally in the absence of full TRF2 dosage. By 3 dpf, however, *terfa* loss unmasks a critical requirement during the onset of stratification: *terfa*^−/−^ larvae exhibit catastrophic failure of lamination, microphthalmia and complete loss of ONL, INL and GCL, whereas *terfa*^+/−^ retinas show thinner layers and reduced cell number despite preserved gross architecture. These defects coincide with fragmentation and shortening of Müller glial processes and a marked reduction in Müller cell number, underscoring TRF2 as an upstream organizer of the Müller scaffold that is essential for neuronal circuit establishment and maintenance. This narrow developmental window and cell-type specificity are difficult to reconcile with a purely canonical telomere-protective role, which would be expected to impair early progenitor proliferation or trigger widespread senescence and apoptosis from the onset of neurogenesis.

The dissociation between telomere-driven genome instability and neurogenic defects further supports a non-canonical function of TRF2 in retinal progenitors and Müller glia. In *terfa*^−/−^ larvae, γH2AX and TIF analyses reveal a genome-wide DDR with telomere-associated and extra-telomeric damage foci, accompanied by massive caspase-3 activation and TUNEL-positive apoptosis, consistent with the established role of TRF2 loss in eliciting telomere uncapping, ATM activation and p53-dependent cell death. In striking contrast, *terfa*^+/−^ retinas display clear defects in lamination, reduced Müller scaffold length and depletion of specific neuronal populations without detectable increases in γH2AX, TIFs or apoptosis. Instead, our transcriptional analyses revealed that both *terfa*^+/−^ and *terfa*^−/−^ zebrafish share a strong down-regulation of phototransduction genes, showing that TRF2 half dosage is sufficient to reprogramme the transcriptional network of developing photoreceptors and retinal neurons in the absence of overt DNA damage. The persistence of key genes of this signature at 6 mpf in *terfa*^+/−^ retinas suggests that early TRF2 insufficiency can imprint on retinal circuits. This dosage separation between lethal genome instability (*terfa*^−/−^) and DDR-independent neurogenic impairment and transcriptional reprogramming (*terfa*^+/−^) mirrors previous findings that zebrafish TRF2 performs DDR-dependent and -independent functions during neurodevelopment and that ATM-dependent DDR explains only part of the embryonic phenotype [9]. Together, these lines of evidence strongly argue that in the retina, TRF2 dosage modulates a developmental programme, particularly within Müller glia and Pax6⁺ retinal progenitors, beyond its classical protective role at chromosome ends.

Several limitations of this study suggest directions for further work. First, while DDR profiling shows a clear separation between genome-wide DNA damage in *terfa*^−/−^ larvae and its absence in *terfa*^+/−^ retinas, direct demonstration that blunting telomere deprotection signalling does not reverse the retinal defects of heterozygous zebrafish is missing. Second, the mechanistic basis of the transcriptional reprogramming remains elusive. Recent reports in mammalian cells show that non-telomeric TRF2 can bind interstitial telomeric sequences (ITS) to regulate neighbouring genes [13] or G-quadruplex-containing promoters enforcing repression of differentiation genes [14]. Whether analogous mechanisms operate at promoters of genes identified here is unknown. Third, the study focuses on zebrafish, which, although an excellent model for retinal development, differs from mammals in cone-rich retinal architecture and regenerative capacity [15]. Testing whether similar TRF2-dependent programmes exist in mouse or human retinal organoids would strengthen translational relevance. Finally, while the persistence of transcriptional alterations at six months argues for long-term consequences of developmental TRF2 insufficiency, functional assays directly linking these changes to age-related visual decline are missing. Longitudinal recordings of behavioural visual tests across the lifespan of *terfa*^+/−^ zebrafish would reveal TRF2 as a driver of retinal ageing rather than a developmental regulator whose effects happen to be long-lasting. Despite these limitations, the present data strongly support a model in which TRF2 operates as a dosage-sensitive, non-canonical regulator of retinal neurogenesis whose early dysfunction imprints a durable vulnerability state.

Our findings also invite a more speculative, systems-level interpretation: that there is an evolutionary advantage in tightly coupling telomere and genome stability to sensory organs, such as eyes and ears, which continuously interrogate the environment. Sensory epithelia experience chronic exposure to light, fluctuating oxygen tension and oxidative stress, conditions known to modulate telomere dynamics and DNA damage signalling, and they rely on glial networks to buffer these stresses and maintain neuronal function. By integrating canonical telomere capping with extra-telomeric control in glial differentiation, TRF2 may link the pace of retinal development with the anticipated lifetime burden of environmental insults. In this hypothesis, subtle shifts in TRF2 levels, whether through genetic variation, early-life stress or stochastic events, could generate retinas that are anatomically similar at juvenile stages yet differ markedly in their resilience to ageing and disease. Interestingly, it was reported that impairment in the five senses predicts future physical dysfunction, cognitive decline, weight loss, frailty and increased mortality, arguing that preserved sensory function is a key component of healthy ageing [16]. We propose that sensory systems are particularly vulnerable to developmental genome-maintenance pathways, as illustrated here for TRF2, and that dissecting these mechanisms in the eye may uncover general principles governing other sensory interfaces with the environment.

## Supplementary Material



## Data Availability

Detailed information on antibodies and primers is listed in supplementary material, table S1. SnRNA-seq DEGs are listed in supplementary material, table S2. All raw sequencing reads for snRNA-seq are available in the NCBI Sequence Read Archive (SRA) under BioProject accession number PRJNA1436113. Bulk RNA-seq DEGs are listed in supplementary material, table S3. All raw sequencing reads for bulk RNA-seq are available in the NCBI Sequence Read Archive (SRA) under BioProject accession number PRJNA1437887. DEGs shared between snRNA-seq retinal cluster 3 and bulk RNA-seq are listed in supplementary material, table S4. Supplementary material is available online [17].

## Theme

One contribution of 21 to a theme issue ‘Developmental circuits in ageing’.
