# Supplementary material for: Non-canonical telomere repeat-binding factor 2 control of retinal development
Source: Philos Trans R Soc Lond B Biol Sci. 2026 Nov 12;381(1963):20250339. doi: 10.1098/rstb.2025.0339 (PMC13755918; doi:10.1098/rstb.2025.0339)
Supplement: Supplementary file 1 [file rstb.2025.0339.zip › rstb.2025.0339/rstb20250339_si_001.pdf]

Non-canonical TRF2 control of retinal development

Ge Gao[1*], Zhen Xie[1*], Jing Wang[2*], Yilin Ying[1], Ruochen Pu[1], Yanping Li[1], Xiaotuo Zhang[1], Eric Gilson[1,3,4†] and Jing Ye[1†]

[1]Department of Geriatrics, Medical Center on Aging of Shanghai Ruijin Hospital, Shanghai Jiaotong University School of Medicine, Shanghai, China; Pole Sino-Francais de Recherches en Sciences du Vivant et Genomique, International Research Project Cancer, Aging and Hematology, Ruijin Hospital, Shanghai; The State Key Laboratory of Medical Genomics, Shanghai;
[2]Department of Ophthalmology, Shanghai General Hospital, Shanghai Jiaotong University School of Medicine, No. 100 Haining Road, Shanghai, 200080 (PR China)
[3]University Côte d'Azur, Inserm, CNRS, IRCAN, Nice, France;
[4]Department of Medical Genetics, IHU RespirERA, CHU, Nice, France.
*These authors contributed equally to this work. †These authors jointly supervised this work.

Supplementary figures

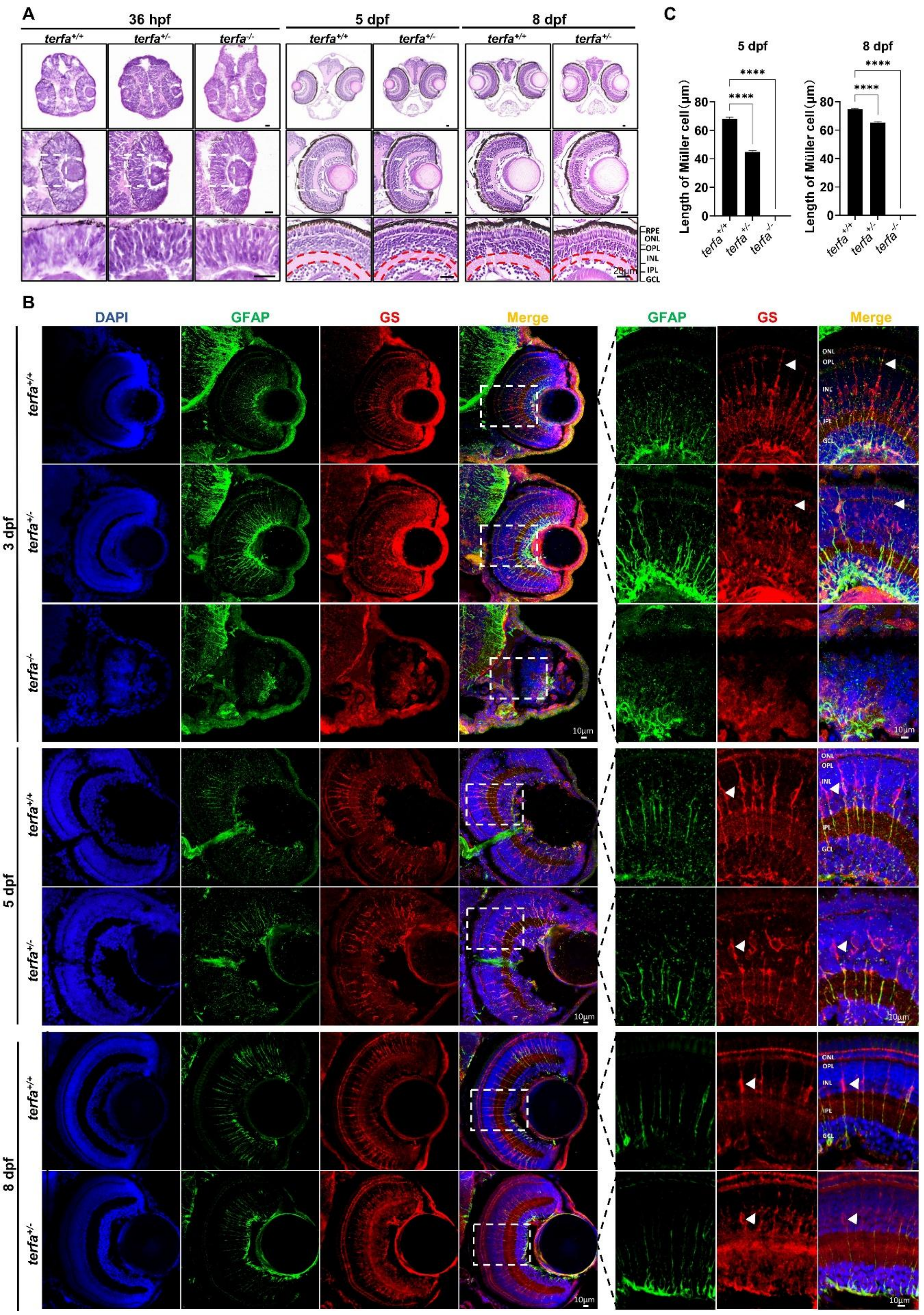
A
36 hpf
5 dpf
8 dpf
terfa+/+
terfa+/-
terfa-/-
RPE
ONL
OPL
INL
IPL
GCL
20μm
B
DAPI
GFAP
GS
Merge
3 dpf
5 dpf
8 dpf
10μm
C
Length of Müller cell (μm)
****
0
20
40
60
80

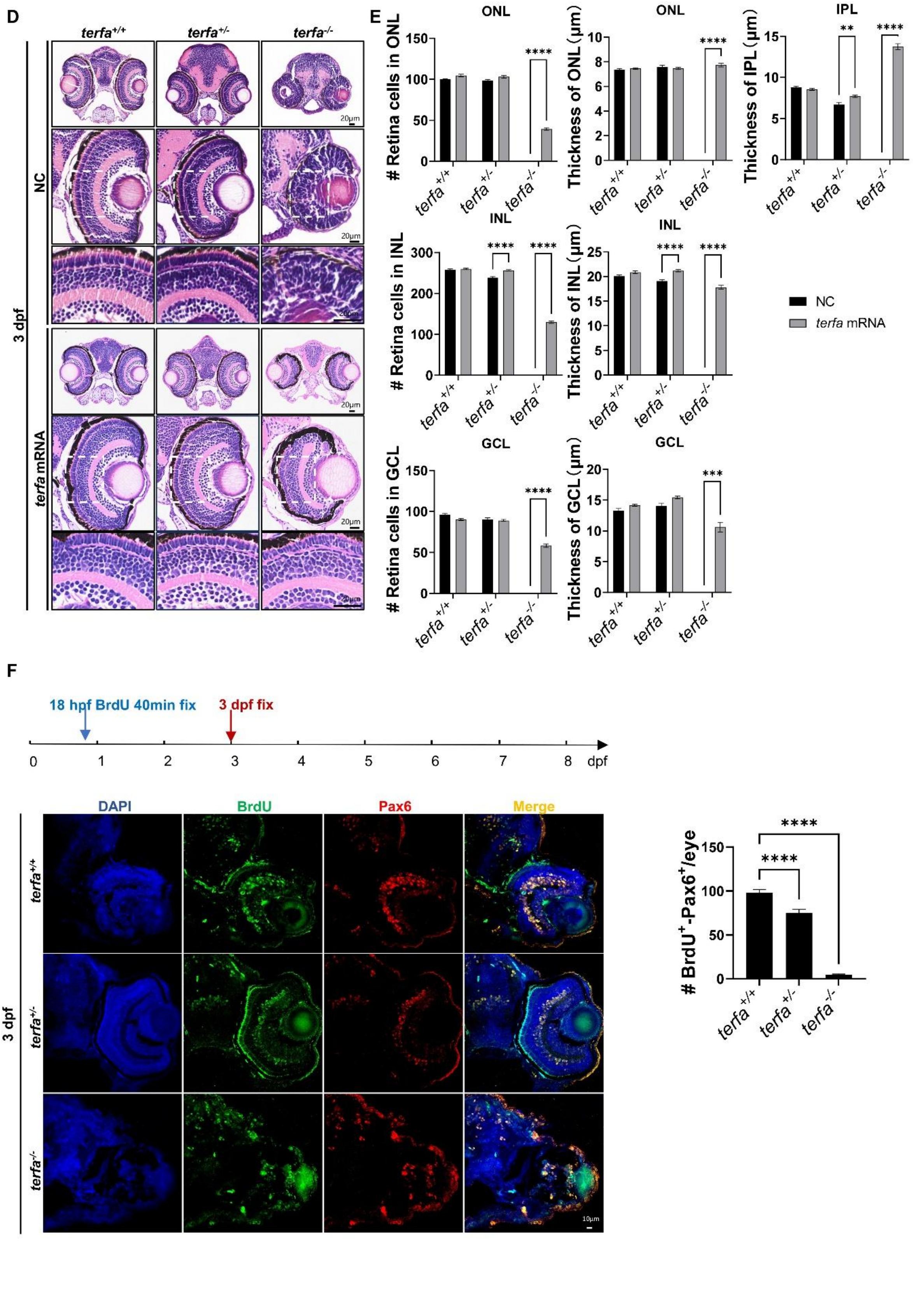
D
terfa+/+
terfa+/-
terfa-/-
NC
terfa mRNA
3 dpf
20μm
E
ONL
Retina cells in ONL
Thickness of ONL (μm)
IPL
Thickness of IPL (μm)
INL
Retina cells in INL
Thickness of INL (μm)
GCL
Retina cells in GCL
Thickness of GCL (μm)
NC
terfa mRNA
F
18 hpf BrdU 40min fix
3 dpf fix
dpf
DAPI
BrdU
Pax6
Merge
10μm
BrdU+-Pax6+/eye

Fig. S1 **TRF2 ablation induces neural developmental failure in the retina.**

(A) Representative transverse H&E sections of 36 hpf (*terfa*$^{+/+}$, *terfa*$^{+/-}$, and *terfa*$^{-/-}$), 5 dpf and 8 dpf retinas (*terfa*$^{+/+}$, *terfa*$^{+/-}$) with magnified ROIs; dashed lines/arrows indicate measurement intervals. *terfa*$^{-/-}$ larvae were absent at 5/8 dpf because of mortality. Scale bar, 20 μm. (B) Representative confocal images of Müller glia at 3 dpf (*terfa*$^{+/+}$, *terfa*$^{+/-}$, and *terfa*$^{-/-}$), 5 dpf and 8 dpf (*terfa*$^{+/+}$, *terfa*$^{+/-}$), stained for GFAP and GS with DAPI. Merge shows GFAP/GS co-localization. Scale bar, 10 μm. (C) Quantification of Müller cell length in 5/8 dpf retina (GCL basal end-feet to ONL apical processes). Up to 10 Müller cells were measured per eye. At 5 dpf, (n=175 cells for *terfa*$^{+/+}$; n=202 cells for *terfa*$^{+/-}$), at 8 dpf, (n=359, 286). *terfa*$^{-/-}$ was counted as 0 at 5/8 dpf (mortality). (D) Representative H&E-stained transverse sections of zebrafish heads/retinas at 3 dpf across the indicated genotypes under negative control conditions (NC; control mRNA injection) and after *terfa* mRNA injection at the one-cell stage (*terfa*). Each condition is shown as a wide-field overview of the head and bilateral eyes (top), magnified central retina (middle), and higher-magnification ROIs (bottom; dashed boxes). Scale bar, 20 μm. (E) Morphometric quantification of retinal cell number (ONL/INL/GCL) and layer thickness (ONL/INL/IPL/GCL) at 3 dpf in NC and *terfa* mRNA injected embryos. NC: n=61 eyes (*terfa*$^{+/+}$), n=43 (*terfa*$^{+/-}$); *terfa*$^{-/-}$ was not quantified for layers due to severely disorganized architecture (set as n=0). *terfa* mRNA: n=46 eyes (*terfa*$^{+/+}$), n=43 (*terfa*$^{+/-}$), n=18 (*terfa*$^{-/-}$). Data are presented as mean ± SEM. Statistical analyses were performed using two-way ANOVA followed by Tukey's multiple comparisons test. (*P < 0.05, **P < 0.01, ***P < 0.001, ****P < 0.0001). (F) BrdU birth-dating of retinal progenitors born at 18 hpf: larvae were pulsed with BrdU at 18 hpf and fixed at 3 dpf for co-staining of BrdU and Pax6 with DAPI. Histogram shows BrdU+/Pax6+ cells per retinal section (n=20 eyes per group). Scale bar, 10 μm. Data are presented as mean ± SEM. Statistical analyses were performed using unpaired two-sided t tests (*P < 0.05, **P < 0.01, ***P < 0.001, ****P < 0.0001).

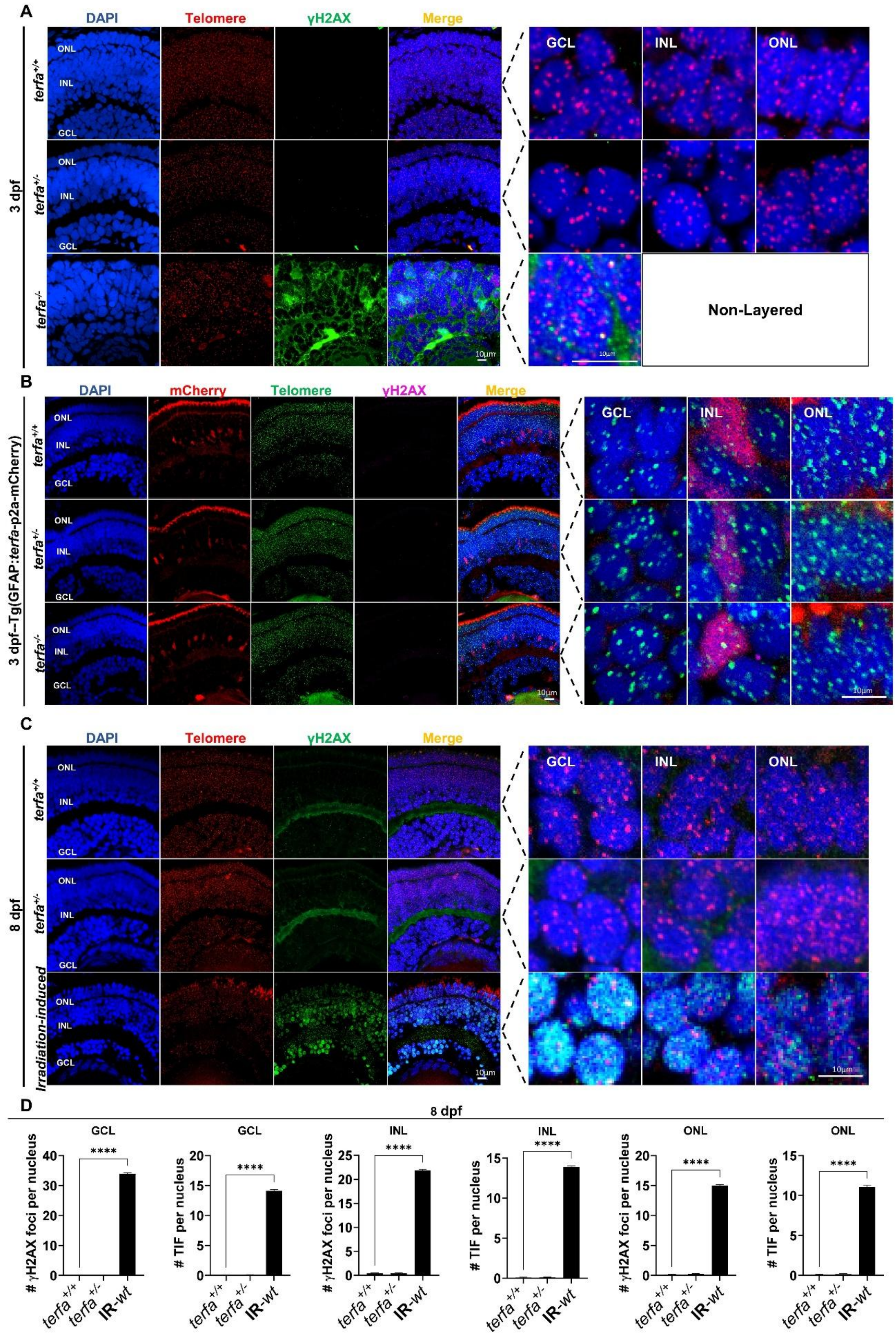

A
DAPI
Telomere
γH2AX
Merge
3 dpf
terfa+/+
terfa+/-
terfa-/-
ONL
INL
GCL
GCL
INL
ONL
Non-Layered
10μm
B
DAPI
mCherry
Telomere
γH2AX
Merge
3 dpf--Tg(GFAP:terfa-p2a-mCherry)
C
DAPI
Telomere
γH2AX
Merge
8 dpf
Irradiation-induced
D
8 dpf
γH2AX foci per nucleus
TIF per nucleus
****
terfa +/+
terfa +/-
IR-wt

E

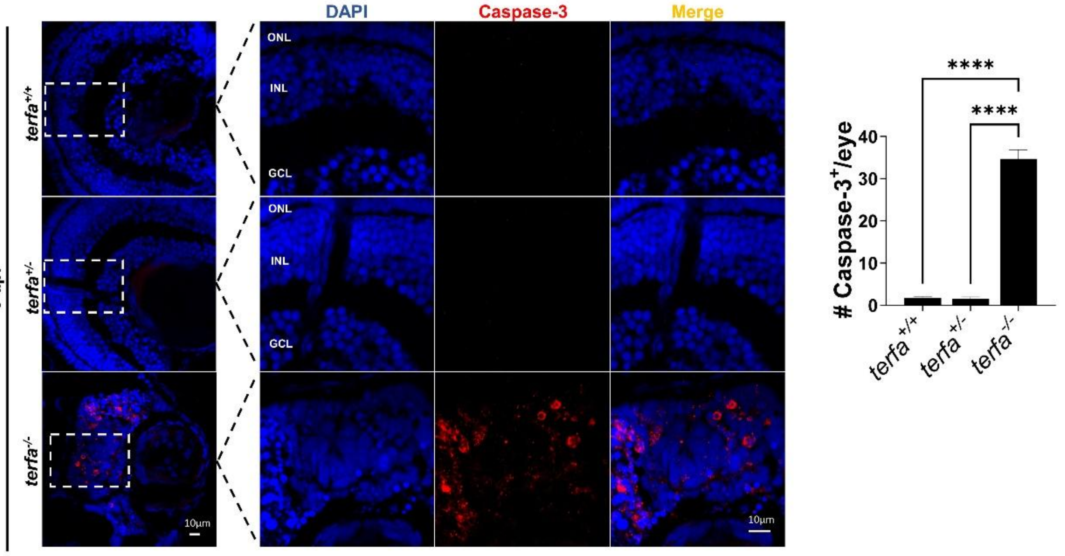

F

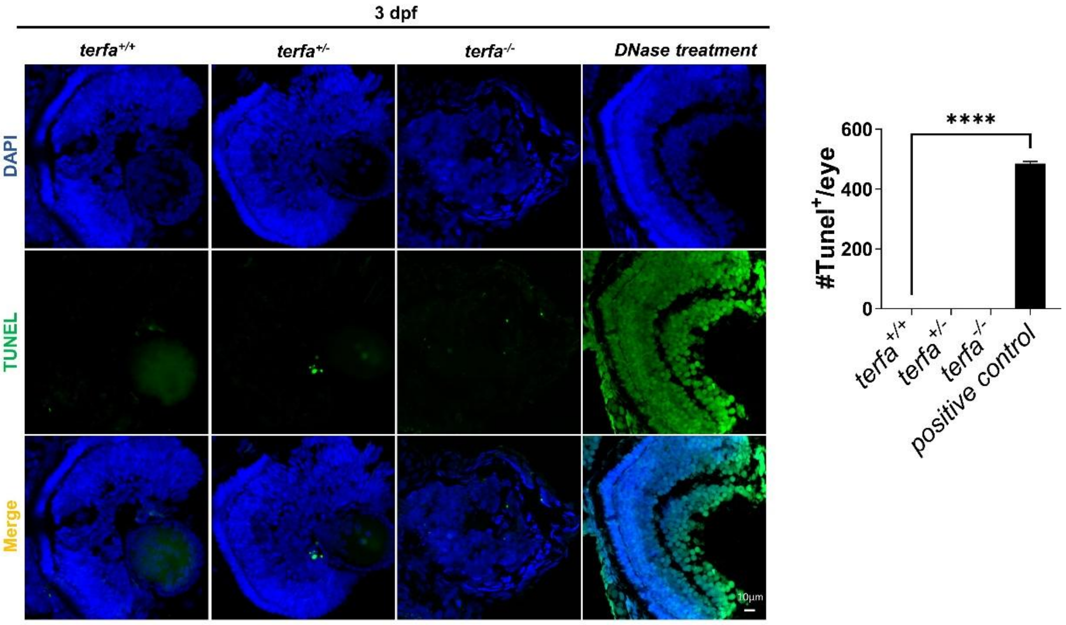

Fig. S2 **TRF2 ablation affected retinal neural development through a dual effect of telomeric & extra-telomeric properties.**

(A) Representative confocal images of 3 dpf transverse retinal sections stained for γH2AX and telomeres with DAPI (blue). Images are shown for *terfa*$^{+/+}$, *terfa*$^{+/-}$, and *terfa*$^{-/-}$. Retinal layers (ONL, INL, GCL) are indicated. Left panels show retinal overviews; dashed boxes/lines mark regions magnified in the right panels, with high-magnification insets for ONL/INL/GCL. Scale

bar, 10 μm. (B) Representative confocal images of 3 dpf transverse retinal sections from Tg(GFAP:*terfa*-p2a-mCherry) zebrafish, shown for *terfa*$^{+/+}$, *terfa*$^{+/-}$, and *terfa*$^{-/-}$. Sections were stained for telomeres (green) and γH2AX (magenta), with DAPI counterstain (blue); mCherry (red) indicates transgene-derived fluorescence. Left panels show retinal overviews with labeled layers (ONL, INL, GCL), and merged images. Dashed boxes indicate regions of interest (ROIs), which are shown at higher magnification in the right panels. High-magnification images display layer-specific views of GCL, INL, and ONL. Scale bar, 10 μm. (C) Representative confocal images of 8 dpf transverse retinal sections stained for γH2AX and telomeres with DAPI (blue). Images are shown for *terfa*$^{+/+}$, *terfa*$^{+/-}$, and irradiation-treated larvae (positive control). Retinal layers (ONL, INL, GCL) are indicated. Left panels show retinal overviews; dashed boxes/lines mark regions magnified in the right panels, with high-magnification insets for ONL/INL/GCL. Scale bar, 10 μm. (D) Layer-specific quantification of γH2AX foci and TIFs per nucleus at 8 dpf in GCL, INL, and ONL for *terfa*$^{+/+}$, *terfa*$^{+/-}$, and irradiation-treated controls. *terfa*$^{-/-}$ was excluded due to post-3-dpf mortality. For each eye, 3–5 cells were randomly counted (n=55 cells for *terfa*$^{+/+}$, n=55 for *terfa*$^{+/-}$, n=97 for irradiation-treated). (E) Representative confocal images of cleaved caspase-3 staining at 3 dpf with DAPI (blue), with dashed boxes indicating regions of interest (ROIs) that are magnified in the right panels, and quantification of caspase-3+ cells per retinal section. n=20 eyes for *terfa*$^{+/+}$ and *terfa*$^{+/-}$; n=18 eyes for *terfa*$^{-/-}$. Scale bar, 10 μm. (F) Representative TUNEL staining at 3 dpf with DAPI (blue); DNase-treated sections served as technical positive controls. Quantification shows TUNEL+ cells per retinal section (n=18 eyes per genotype; n=12 eyes for positive control). Scale bar, 10 μm.

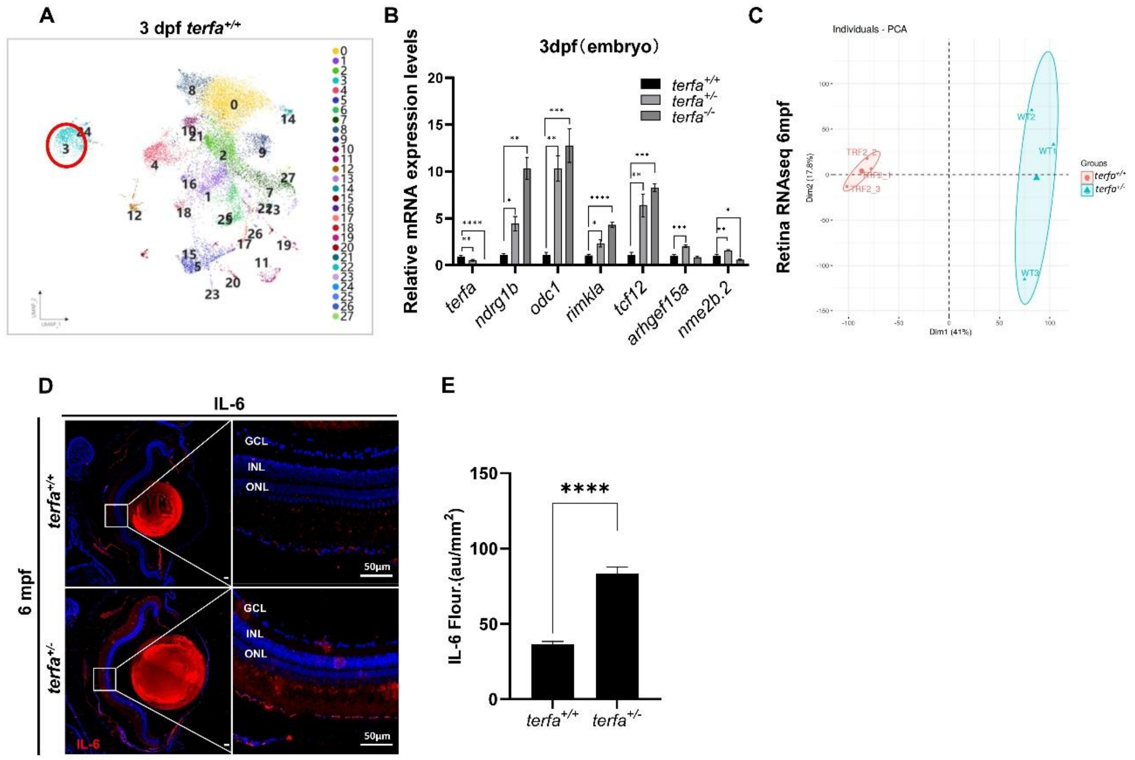

Fig. S3 **TRF2 ablation leads to retinal transcriptional reprogramming.**

(A) UMAP projections of 3 dpf *terfa*$^{+/+}$ larval snRNA-seq data. Cluster 3 (putative retinal lineage; red circles). (B) RT–qPCR validation of selected candidates (including *terfa, ndrg1b*, *odc1, rimkla,* etc.) in dissected head tissues from 3 dpf *terfa*$^{+/+}$, *terfa*$^{+/-}$ and *terfa*$^{-/-}$ zebrafish, presented as relative mRNA expression (mean ± SEM). Statistics: *P<0.05, **P<0.01.(C) PCA of bulk RNA-seq from 6 mpf retinas showing separation between *terfa*$^{+/-}$ (TRF2; red circles) and *terfa*$^{+/+}$ (WT; blue triangles) (n = 3 biological replicates per group). Dim1 and Dim2 explain 41% and 17.8% of variance, respectively. (D) Representative immunofluorescence images of 6 mpf zebrafish retinal sections stained for IL-6 (red) with DAPI counterstain (blue), shown for *terfa*$^{+/+}$ and *terfa*$^{+/-}$. Left panels show low-magnification views of the whole eye, with boxed regions indicating areas magnified in the right panels. (E) Quantification of IL-6 fluorescence intensity in retinal sections from 6 mpf *terfa*$^{+/+}$ and *terfa*$^{+/-}$ zebrafish, presented as fluorescence per unit area (a.u./mm²). Data are presented as mean ± SEM (n=20 eyes per

genotype). Statistical analyses were performed using unpaired two-sided t tests (****$P < 0.0001$).
